# Influenza Vaccination Preferences Among Adults Aged 60 Years and Older in Beijing, China

**DOI:** 10.1001/jamanetworkopen.2026.38674

**Published:** 2026-10-12

**Authors:** Mengmeng Jia, Yuxi Liu, Jiarui Zheng, Fenghua Su, Jitao Zhang, Mingyue Jiang, Tongsiman Wang, Xinqi Wang, Tao Wang, Wei Dai, Luzhao Feng

**Affiliations:** 1 Key Laboratory of Pathogen Infection Prevention and Control (Peking Union Medical College), Ministry of Education, National Institute of Pathogen Biology, Chinese Academy of Medical Sciences and Peking Union Medical College, Beijing, China; 2 School of Population Medicine and Public Health, Chinese Academy of Medical Sciences & Peking Union Medical College, State Key Laboratory of Respiratory Health and Multimorbidity, Public Health Emergency Management Innovation Center of Beijing Higher Education Innovation Center for Philosophy and Social Sciences, Beijing, China; 3 Beijing Changping District Center for Disease Control and Prevention (Changping District Health Supervision Institute), Beijing, China; 4 Beijing Dongcheng Center for Disease Control and Prevention, Beijing, China; 5 Yanjing Medical College, Capital Medical University, Beijing, China

## Abstract

**Importance:**

Influenza vaccination coverage among older adults remains suboptimal in China despite free vaccination policies in some regions. Understanding their preferences may inform strategies to improve uptake.

**Objective:**

To quantify preferences for influenza vaccination attributes and assess preference heterogeneity by prior influenza vaccination history.

**Design, Setting, and Participants:**

This community-based survey study was conducted at 2 districts in Beijing, China, where influenza vaccination is provided free of charge for adults aged 60 years or older. A convenience sample of adults aged 60 years or older was included from 6 Beijing subdistricts and participated in a discrete choice experiment conducted from August to September 2025.

**Main Outcomes and Measures:**

Stated choice among 2 hypothetical influenza vaccination profiles and an opt-out option. Attributes included vaccine effectiveness, risk of mild adverse effects, vaccination venue and operating hours, outreach method, information source, and message content. Conditional logit, mixed logit, scenario simulations, and vaccination-history interaction analyses were performed.

**Results:**

Among 514 participants included in the analyses, the median age was 71 years (IQR, 65-79 years), and 356 (69.3%) were women. Higher vaccine effectiveness was associated with greater vaccination preference (β = 2.12; 95% CI, 1.37 to 2.88), whereas higher perceived risk of mild adverse effects was associated with lower preference (β = −5.53; 95% CI, −7.83 to −3.22). Participants preferred community-based vaccination settings, telephone reminders (β = 1.06; 95% CI, 0.76 to 1.36), information from government or community organizations (β = 0.78; 95% CI, 0.54 to 1.02), and messages emphasizing protection of family members (β = 0.50; 95% CI, 0.26 to 0.74). Increasing vaccine effectiveness from 20% to 80% and reducing perceived risk of adverse effects from 20% to 5% were associated with 30.74– and 19.96–percentage-point (pp) increases in the model-estimated choice probability, respectively. Compared with participants with prior vaccination, never-vaccinated participants had a greater preference for opting out (difference in β = 1.07; 95% CI, 0.11 to 2.03), greater sensitivity to mild adverse effect risk (difference in β per 10-pp increase, −0.49; 95% CI, −0.93 to −0.05), and lower responsiveness to vaccine effectiveness (difference in β per 10-pp increase, −0.18; 95% CI, −0.31 to −0.04).

**Conclusions and Relevance:**

In this discrete choice experiment of older adults in Beijing, influenza vaccination preferences were associated with vaccine profiles, trusted communication, and community-based service delivery. Prior vaccination history identified differences in benefit-risk weighting that may inform broad and targeted vaccination strategies.

## Key Points

**Question:**

What factors shape influenza vaccination preferences among older adults in Beijing, China, where influenza vaccines are provided for free?

**Findings:**

In this survey study of 514 older adults, higher vaccine effectiveness, lower perceived risk of mild adverse effects, community-based vaccination, telephone reminders, government or community information sources, and family-protection messages were associated with greater vaccination preference. Never-vaccinated adults showed greater opt-out preference, greater sensitivity to the risk of adverse effects, and weaker responsiveness to vaccine effectiveness.

**Meaning:**

These findings may inform vaccination strategies that integrate accessible services, trusted communication, and tailored approaches based on vaccination history.

## Teaser

This survey study investigates influenza vaccination preferences among older adults in China to consider strategies for improved adoption.

## Introduction

Seasonal influenza remains an important public health challenge, causing substantial morbidity and mortality worldwide, particularly among older adults and individuals with chronic conditions.^1^ Influenza vaccination is an effective strategy for reducing influenza-related infection, severe illness, and complications in these high-risk populations.^2,3,4^ However, influenza vaccination coverage among adults remains suboptimal in many countries, including China. A meta-analysis reported a pooled influenza vaccination rate of 17% (95% CI, 14%-21%) in China, with lower rates observed among individuals with chronic diseases (13%) and in areas without free vaccination policies (7%).^5^

Beijing was one of the first cities in China to provide free influenza vaccination for adults aged 60 years or older, beginning in 2007. Although this policy improved vaccine access and increased uptake to approximately 50% during 2011 to 2015, compared with 11.4% to 14.9% observed in self-funded vaccination areas, coverage among older adults has remained suboptimal.^6^ These findings suggest that removing financial barriers alone may be insufficient and that vaccination decisions may continue to be shaped by vaccine confidence, service accessibility, communication channels, and social influences. The World Health Organization’s Behavioral and Social Drivers (BeSD) framework conceptualizes vaccine uptake as being influenced by thinking and feeling, social processes, motivation, and practical issues, providing a useful framework for identifying modifiable factors of vaccination behavior.^7,8,9,10^

Discrete choice experiments (DCEs) can quantify preferences and trade-offs of different attributes in health care decision-making.^11^ Although DCEs have been increasingly applied to vaccination preferences,^12,13^ evidence remains limited regarding how older adults in publicly funded vaccination settings weigh vaccine characteristics, service-delivery features, and communication-related attributes. Evidence is also limited on preference heterogeneity across subgroups of older adults.^14^

We conducted a DCE to quantify how older adults in a publicly funded program valued vaccine characteristics, service-delivery features, and communication attributes. We hypothesized that preferences would vary with vaccine effectiveness, safety, service accessibility, outreach methods, information sources, message content, and a history of influenza vaccination.

## Methods

### Study Design, Setting, Participants, and Ethics

A community-based DCE was conducted between August and September 2025 in Dongcheng and Changping Districts, Beijing, China. A convenience sample of adults aged 60 years or older was recruited from 6 subdistricts, including 3 in each district. The participating communities were selected in coordination with the district-level Centers for Disease Control and Prevention based on feasibility and willingness to participate rather than through random sampling. The district Centers for Disease Control and Prevention coordinated with local community health service centers to invite potentially eligible older residents to participate. Because the number of individuals invited or screened was not systematically recorded, a participation rate could not be calculated.

The reporting of this survey-based DCE was guided by relevant American Association for Public Opinion Research (AAPOR) recommendations for survey studies and the Discrete Choice Experiment (DIRECT) reporting checklist.^15,16^ Key reporting elements associated with survey procedures and DCE methods are summarized in eTables 1 and 2 in Supplement 1. The study was approved by the Ethics Committee of the National Institute of Pathogen Biology, Chinese Academy of Medical Sciences and Peking Union Medical College, Beijing, China (Approval ID: IPB-2025-30). Written informed consent was obtained from all participants before the survey administration.

Eligible participants were aged 60 years or older, provided informed consent, and could complete the survey independently or with standardized assistance. Participants received a nonmonetary umbrella after completion. The sample size was estimated using Orme formula,^17^ according to which N ≥ 500*c* / (*t* × a), where t is the number of choice tasks, a is the number of alternatives, and c is the largest number of levels for any attribute. Based on 8 experimental choice tasks, 2 vaccination alternatives per task, and a maximum of 4 levels for an attribute, the minimum required sample size was 125.

The anonymous questionnaire was administered in Chinese using standardized tablet computers. The electronic interface was adapted for older adults through enlarged font sizes, simplified layout, and clear visual presentation. Participants completed the survey independently whenever possible; trained research staff provided standardized assistance with tablet use or clarification of question wording when needed, without influencing participants’ choices. The survey instrument involved 5 sections: (1) an introductory section and the informed consent form; (2) sociodemographic questions; (3) introduction of DCE with 1 practice choice question; (4) 8 choice tasks with 1 additional repeated consistency check task; and (5) questions about attitudes toward and practices associated with influenza vaccination.

### Attributes and Levels

Attribute selection was guided by the WHO’s BeSD framework, which highlights practical issues, social processes, and thinking and feeling as key factors of vaccination behavior. Since this study aimed to identify actionable strategies in a setting where vaccination was already provided free of charge, we prioritized attributes that could potentially be modified through vaccination service design, outreach, and risk communication. Vaccine effectiveness and perceived risk of mild adverse effects were additionally included to represent the fundamental benefit-risk considerations underlying vaccine acceptance. Candidate attributes and levels were developed through a literature review, consultation with 5 experts, and pretesting with 5 older adults. The final attributes were refined to ensure theoretical and policy relevance, comprehensibility, and feasibility within a choice task, while the levels reflected realistic current or potentially implementable alternatives.

The final design included 7 attributes covering vaccine profile (perceived risk of mild adverse effects and vaccine effectiveness), practical issues (vaccination location and operating hours), social processes (outreach method and information source), and thinking and feeling (message content). The attributes and levels wording used in this study are listed in Table 1, while the detailed rationales and sources for each attribute and level are provided in eTable 3 in Supplement 1.

**Table 1.** Attributes and Levels Included in the Discrete Choice Experiment

Attributes and levels	Description
**Location for vaccination (vaccination venue)**	
Practical issues	
Level 1	Community health care station
Level 2	Community health care center
Level 3	General hospital
Level 4	Private hospital
**Operating hours (available time slots for influenza vaccination)**	
Practical issues	
Level 1	9 am-5 pm on weekdays
Level 2	5 pm-9 pm on weekdays
Level 3	9 am-12 pm on weekends
Level 4	1 pm-5 pm on weekends
**Outreach method (method used to communicate vaccination information or reminders)**	
Social processes	
Level 1	Telephone call
Level 2	Short message
Level 3	Poster
Level 4	WeChat
**Information source (primary source of information about influenza vaccination that they consider trustworthy)**	
Social processes	
Level 1	Social media
Level 2	Health care workers
Level 3	Government or community
Level 4	Family members and friends
**Message content (promotional message content that older adults find most persuasive or relatable)**	
Thinking and feeling	
Level 1	Getting vaccinated can prevent influenza infection
Level 2	Getting vaccinated can protect family members
Level 3	Not getting vaccinated may result in severe infection
**Risk of mild adverse effects (probability of experiencing mild, short-term adverse effects, such as pain at the injection site)** ^a^	
Vaccine profile	
Level 1	5%
Level 2	10%
Level 3	15%
Level 4	20%
**Vaccine effectiveness (probability that vaccination prevents influenza infection)** ^a^	
Vaccine profile	
Level 1	20%
Level 2	40%
Level 3	60%
Level 4	80%

^a^ Vaccine effectiveness and adverse-effect risk were included as a separate vaccine profile domain.

In each choice task, every attribute level was presented using both an icon and its complete Chinese text description. The icons were intended to reduce reading burden and facilitate comparisons between alternatives rather than to replace the written information. The same icon-text combinations were used consistently throughout the survey and were reviewed during expert consultation and pretesting for conceptual consistency, clarity, distinguishability, and potential misinterpretation. The visual presentation was revised and standardized based on this feedback.

### Experimental Design

A bayesian D-efficient design for estimating main effects was generated using Ngene software (ChoiceMetrics). The design included 24 choice tasks divided into 3 blocks, with each participant completing 1 block containing 8 choice tasks and 1 additional repeated consistency check task. Each task included 2 unlabeled hypothetical vaccination alternatives and 1 opt-out option. An example choice task is shown in the eFigure in Supplement 1. When making choices, respondents were asked to consider all 7 attributes as shown in Table 1. The order of choice tasks and the profiles of vaccine A and B were randomized to minimize response bias.

### Statistical Analyses

Descriptive statistics were used to summarize participant characteristics. Continuous variables are presented as means (SDs) or medians (IQRs), as appropriate, and categorical variables as frequencies (percentages). Differences according to vaccination history were assessed using χ^2^ tests. No sampling weights were applied given that the convenience sampling approach was used. Participants included in the primary analysis had complete data for the DCE choice tasks and variables used in the primary preference models.

A conditional logit model with participant-clustered standard errors was first used to estimate average preferences for influenza vaccination attributes. A mixed logit model was subsequently estimated using simulated maximum likelihood to account for repeated choices within participants and unobserved preference heterogeneity. A sensitivity analysis additionally accounted for potential differences across recruitment subdistricts using subdistrict fixed effects. Estimated mean coefficients from the mixed logit model were used as the primary preference estimates and as the basis for the relative attribute importance, trade-off, and scenario simulation analyses.

Relative attribute importance was calculated using the range of utility across attribute levels. Trade-offs were quantified using marginal rates of substitution. Scenario simulations were conducted to estimate changes in model-estimated choice probabilities under hypothetical vaccination profiles.

Preference heterogeneity was primarily assessed according to influenza vaccination history, and the between-group differences were formally tested with a pooled mixed logit model that included interaction terms between never-vaccinated status and each attribute-level variable. Additional subgroup and latent class analyses were considered exploratory.

All analyses were 2-sided, with statistical significance defined as *P* < .05. Data management and quality control procedures were conducted using SAS version 9.4 (SAS Institute Inc.). Statistical models and scenario simulations were conducted using Stata version 17.0 (StataCorp LLC). R version 4.6.0 (R Foundation for Statistical Computing) was used for data visualization and figure production. Additional details of the statistical analysis are shown in eMethods in Supplement 1.

## Results

### Participant Characteristics

Of 593 participants who completed the survey, 79 failed the consistency check and were excluded. Among the 514 participants included in the final analysis, the median age was 71 years (IQR, 65-79 years), and 356 (69.26%) were female. Overall, 252 (49.03%) had never been vaccinated against influenza (Table 2).

**Table 2.** Participant Characteristics by Prior Influenza Vaccination Status

Characteristics	Total (N = 514)	Ever vaccinated (n = 262)	Never vaccinated (n = 252)	*P* value
Age, y				
Median (IQR)	71 (65-79)	74 (67-82)	69 (63-74)	<.001
≤70 y	236 (45.91)	93 (35.50)	143 (56.75)	
>70 y	278 (54.09)	169 (64.50)	109 (43.25)	
Sex				
Male	158 (30.74)	88 (33.59)	70 (27.78)	.15
Female	356 (69.26)	174 (66.41)	182 (72.22)	
Education level				
Junior high school or below	241 (46.89)	108 (41.22)	133 (52.78)	<.001
High school or technical school	149 (28.99)	68 (25.95)	81 (32.14)	
College and above	124 (24.12)	86 (32.82)	38 (15.08)	
Living arrangement				
Living alone	57 (11.09)	26 (9.92)	31 (12.30)	<.001
Living with family members	355 (69.07)	149 (56.87)	206 (81.75)	
Living in a nursing home	102 (19.84)	87 (33.21)	15 (5.95)	
Monthly disposable income, CNY				
<3000	137 (26.65)	60 (22.90)	77 (30.56)	<.001
3000-4999	112 (21.79)	44 (16.79)	68 (26.98)	
5000-6999	160 (31.13)	77 (29.39)	83 (32.94)	
≥7000	105 (20.43)	81 (30.92)	24 (9.52)	
Any chronic condition				
No	373 (72.57)	201 (76.72)	172 (68.25)	.03
Yes	141 (27.43)	61 (23.28)	80 (31.75)	
Self-reported health status				
Very healthy	72 (14.01)	36 (13.74)	36 (14.29)	.95
Relatively healthy	228 (44.36)	119 (45.42)	109 (43.25)	
Average	161 (31.32)	81 (30.92)	80 (31.75)	
Relatively unhealthy	43 (8.37)	22 (8.40)	21 (8.33)	
Very unhealthy	10 (1.95)	4 (1.53)	6 (2.38)	
Influenza infection since July 2024				
Yes	67 (13.04)	40 (15.27)	27 (10.71)	.12
No	447 (86.96)	222 (84.73)	225 (89.29)	
Most recent influenza vaccination				
Since July 2024	161 (31.32)	161 (61.45)	NA	NA
Before July 2024	101 (19.65)	101 (38.55)	NA	
Never vaccinated	252 (49.03)	NA	252 (100)	

Abbreviations: CNY, Chinese Yuan; NA, not applicable.

### Estimates of Preferences for Influenza Vaccination Attributes

Compared with the conditional logit model, the mixed logit model demonstrated better model fit (eTable 4 in Supplement 1) and was used as the primary model for subsequent analyses. Estimates from the conditional logit and mixed logit models were generally consistent, while the mixed logit model allowed selected preference coefficients to vary across participants.

In the mixed logit model, higher vaccine effectiveness was associated with greater vaccination preference (β = 2.12; 95% CI, 1.37 to 2.88), whereas higher perceived risk of mild adverse effects was associated with lower preference (β = −5.53; 95% CI, −7.83 to −3.22). Compared with vaccination at community health care centers, vaccination at general hospitals (β = −1.12; 95% CI, −1.41 to −0.82) and private hospitals (β = −4.70; 95% CI, −5.39 to −4.01) was less preferred. Among outreach methods, compared with posters, telephone calls had the most positive associations with vaccination preference (β = 1.06; 95% CI, 0.76 to 1.36), followed by WeChat reminders (β = 0.41; 95% CI, 0.14 to 0.69). Compared with social media, information delivered by government or community organizations was significantly preferred (β = 0.78; 95% CI, 0.54 to 1.02). In addition, compared with messages emphasizing personal protection, messages emphasizing protection of family members were also associated with greater preference (β = 0.50; 95% CI, 0.26 to 0.74). Complete estimates for all attribute levels are presented in Table 3. Sensitivity analysis accounting for recruitment subdistrict fixed effects presented qualitatively consistent preference patterns, although baseline vaccination choice varied across subdistricts (eTable 5 in Supplement 1).

**Table 3.** Conditional Logit and Mixed Logit Estimates of Preferences for Influenza Vaccination Attributes

Preference	Conditional logit results		Mixed logit results	
	β (95% CI)	*P* value	β (95% CI)	*P* value
Opt-out	0.68 (0.38 to 0.97)	<.001	0.04 (−0.45 to 0.54)	.86
Outreach method				
Poster	0 [Reference]	NA	0 [Reference]	NA
Short message	0.16 (0.02 to 0.30)	.03	0.28 (−0.02 to 0.58)	.06
Telephone call	0.59 (0.44 to 0.73)	<.001	1.06 (0.76 to 1.36)	<.001
WeChat	0.36 (0.22 to 0.49)	<.001	0.41 (0.14 to 0.69)	<.001
Location for vaccination				
Community health care center	0 [Reference]	NA	0 [Reference]	NA
Community health care station	0.04 (−0.06 to 0.14)	.41	0.08 (−0.15 to 0.31)	.52
General hospital	−0.45 (−0.59 to −0.31)	<.001	−1.12 (−1.41 to −0.82)	<.001
Private hospital	−1.76 (−1.95 to −1.56)	<.001	−4.70 (−5.39 to −4.01)	<.001
Operating hours				
9 am-5 pm on weekdays	0 [Reference]	NA	0 [Reference]	NA
5 pm-9 pm on weekdays	−0.09 (−0.21 to 0.02)	.11	−0.11 (−0.35 to 0.13)	.36
9 am-12 pm on weekends	0.14 (−0.01 to 0.29)	.06	0.19 (−0.09 to 0.47)	.19
1 pm-5 pm on weekends	0.10 (−0.04 to 0.24)	.16	0.09 (−0.18 to 0.36)	.52
Information source				
Social media	0 [Reference]	NA	0 [Reference]	NA
Family members and friends	0.11 (−0.01 to 0.23)	.08	0.02 (−0.21 to 0.24)	.88
Health care workers	0.07 (−0.05 to 0.19)	.28	0.26 (0.01 to 0.51)	.04
Government or community	0.42 (0.30 to 0.53)	<.001	0.78 (0.54 to 1.02)	<.001
Message content				
Getting vaccinated can prevent influenza infection	0 [Reference]	NA	0 [Reference]	NA
Not getting vaccinated may result in severe infection	−0.04 (−0.14 to 0.06)	.40	−0.02 (−0.25 to 0.21)	.84
Getting vaccinated can protect family members	0.24 (0.13 to 0.35)	<.001	0.50 (0.26 to 0.74)	<.001
Risk of mild adverse effects^a^	−2.11 (−3.01 to −1.22)	<.001	−5.53 (−7.83 to −3.22)	<.001
Vaccine effectiveness^a^	1.62 (1.38 to 1.85)	<.001	2.12 (1.37 to 2.88)	<.001

Abbreviation: NA, not applicable.

^a^ Risk of mild adverse effects and vaccine effectiveness were modeled as continuous variables scaled from 0.05 to 0.20, and 0.20 to 0.80, respectively.

### Relative Importance, Scenario Simulations, and Trade-Offs

Relative attribute importance estimates were design-dependent and reflected the utility range represented by the selected attribute levels. Within the experimental design, vaccination location had a utility range of 4.78, accounting for 50.04% of the total utility range, largely associated with aversion to private hospital vaccination. Vaccine effectiveness (1.27 [13.35%]), outreach method (1.06 [11.14%]), perceived adverse-effect risk (0.83 [8.69%]), information source (0.78 [8.17%]), and message content (0.52 [5.49%]) also contributed to preference variation, whereas operating hours contributed least (0.30 [3.14%]).

In single-attribute scenario simulations, increasing vaccine effectiveness from 20% to 80% was associated with the largest increase in model-estimated vaccination choice probability (30.74 percentage points [pp]), followed by replacing poster-based outreach with telephone calls (25.72 pp), and reducing perceived risk of mild adverse effects from 20% to 5% (19.96 pp). Specifying vaccination at a private hospital was less preferred than at health care centers (−32.20 pp) (Figure 1A). In sequential scenario simulations, the baseline profile yielded a model-estimated choice probability of 32.64%. Adding favorable vaccine-profile attributes produced the largest increase, to 79.86%, followed by smaller increases after adding practical-accessibility, social-process, and thinking-and-feeling components, resulting in a final estimated probability of 98.17% (Figure 1B).

**Figure 1.**
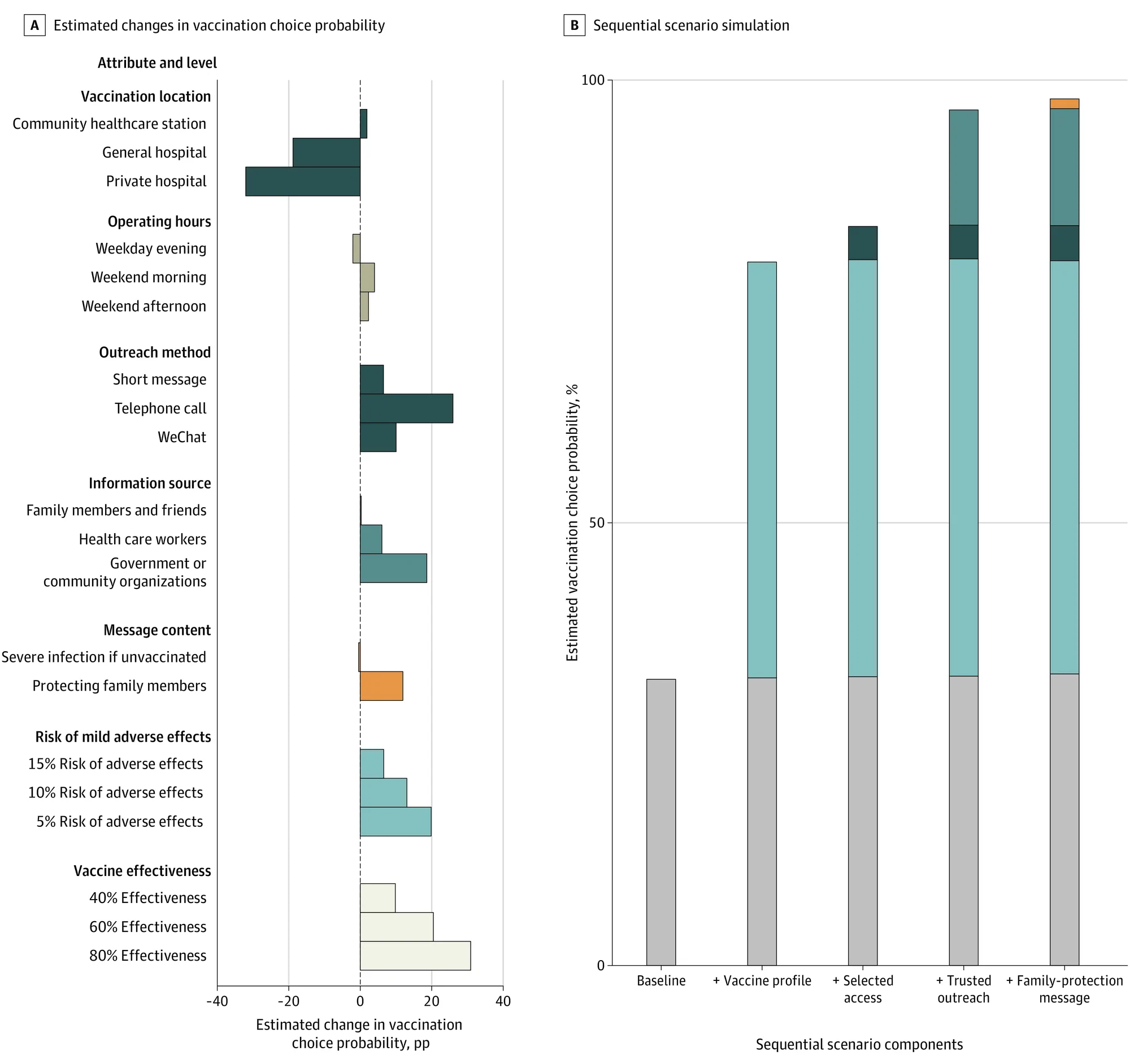
Bar Charts of Model-Estimated Changes in Influenza Vaccination Choice Probability Under Alternative Attribute and Sequential Scenarios A, The baseline profile included vaccination at a community health care center during weekday working hours, poster-based outreach, information from social media, a message emphasizing prevention of influenza infection, a 20% risk of mild adverse effects, and 20% vaccine effectiveness. B, Sequential scenario simulation showing the estimated vaccination choice probability as preferred components were cumulatively added to the baseline profile. The vaccine-profile scenario combined a 5% risk of mild adverse effects and 80% vaccine effectiveness; selected access added vaccination at a community health care station and weekend morning operating hours; trusted outreach added telephone outreach and information from government or community organizations; and the family-protection scenario added a message emphasizing protection of family members. pp indicates percentage point.

In trade-off analyses, a 5.0-pp increase in the perceived risk of adverse effects was utility-equivalent to a 13.0-pp increase in vaccine effectiveness. Telephone reminders and government or community information sources were equivalent to 50.1- and 36.7-pp increases in vaccine effectiveness, respectively (eTable 6 in Supplement 1).

### Preference Heterogeneity

Preference patterns were generally similar according to vaccination history, but significant differences were observed for 4 coefficients (Figure 2; eTable 7 in Supplement 1). Participants who had never received influenza vaccination had a greater preference for opting out than those with prior vaccination (difference in β = 1.07; 95% CI, 0.11 to 2.03).

**Figure 2.**
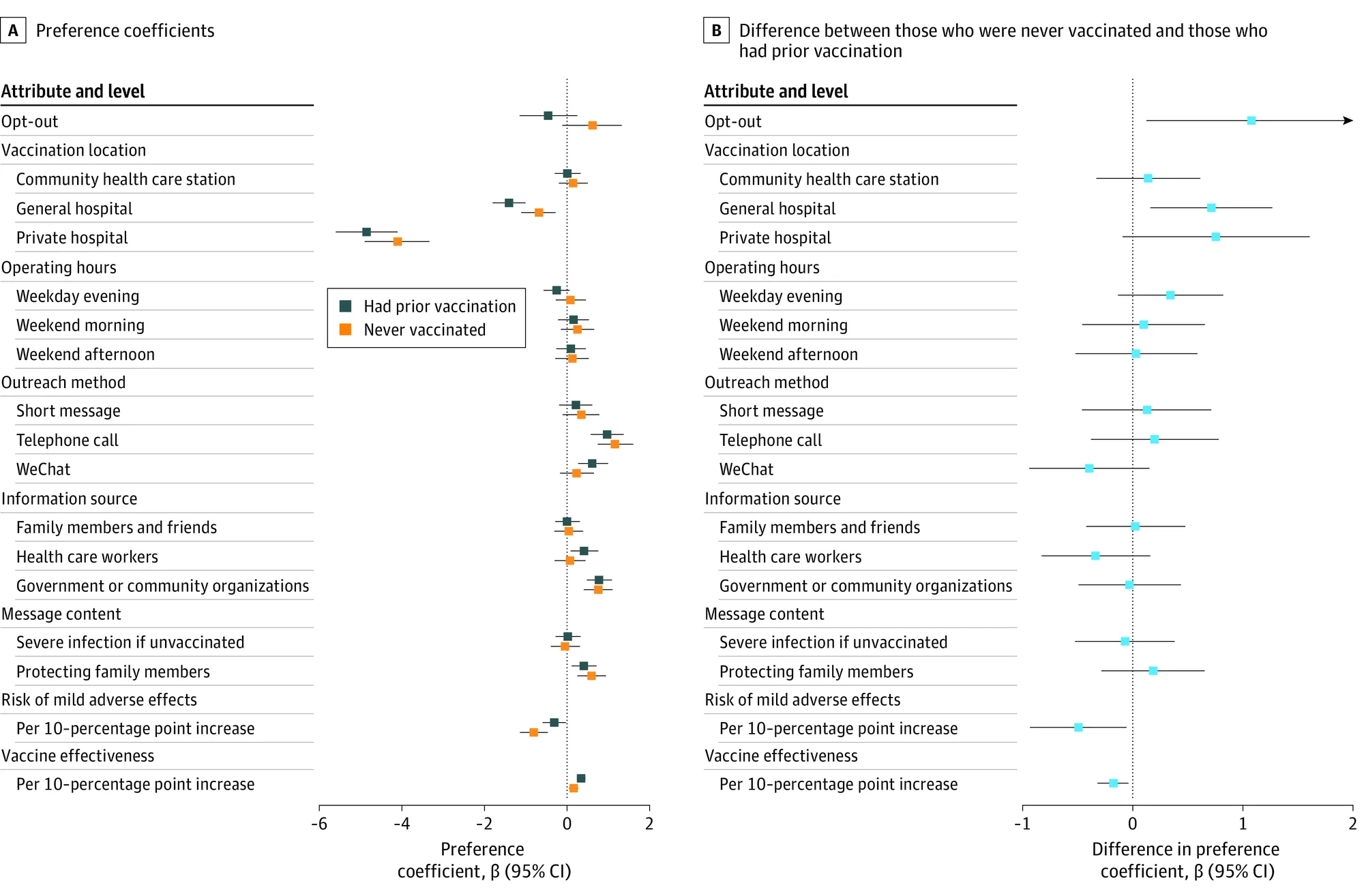
Dot Plot of Influenza Vaccination Preferences According to Vaccination History A, Preference coefficients and 95% CIs among participants with prior influenza vaccination and those who had never received influenza vaccination. Positive coefficients indicate greater preference and negative coefficients indicate lower preference relative to the corresponding reference level. Coefficients were derived from the pooled mixed logit model with interactions between prior vaccination history and each model term. B, Differences in preference coefficients between participants who had never received influenza vaccination and those with prior vaccination. Positive values indicate a more positive, or less negative, coefficient among participants who had never been vaccinated. Error bars indicate 95% CIs. Coefficients for risk of mild adverse effects and vaccine effectiveness are expressed per 10–percentage-point increase.

Although general hospitals were negatively valued in both groups, this preference was less negative among participants who had never been vaccinated (difference in β = 0.71; 95% CI, 0.16 to 1.26). Never-vaccinated participants were more sensitive to increases in the risk of mild adverse effects (difference in β per 10-pp increase, −0.49; 95% CI, −0.93 to −0.05) and less responsive to increases in vaccine effectiveness (difference in β per 10-pp increase, −0.18; 95% CI, −0.31 to −0.04). Differences for the other vaccination and service attributes were not statistically significant. Separately estimated stratified models showed broadly similar patterns (eTable 8 in Supplement 1).

Exploratory subgroup analyses showed broadly consistent directions of preference estimates (eTables 9-12 in Supplement 1). In addition, exploratory latent class analysis identified 3 classes that differed in responsiveness to outreach and in the relative weight placed on perceived risk of mild adverse effects and vaccine effectiveness (eTable 13 in Supplement 1).

## Discussion

In this discrete choice experiment among older adults in Beijing, influenza vaccination preferences were associated with vaccine profile characteristics, community-based service delivery, and trusted personalized communication, even in a setting where vaccination was provided free of charge. Participants preferred higher vaccine effectiveness, lower risk of mild adverse effects, community-based vaccination settings over hospital settings, telephone reminders, information from government or community organizations, and messages emphasizing protection of family members. These findings suggest that factors beyond financial access were associated with vaccination preferences among older adults.

Even in a setting where vaccination was free of charge, perceptions of vaccine effectiveness and safety remained important considerations in vaccination preference. These findings are particularly relevant as China expands financial support for influenza vaccination, including allowing the use of personal medical insurance accounts to pay for influenza vaccines.^18^ The apparent preferences for higher effectiveness and lower adverse-effect risk suggest that vaccine confidence remains central even when financial barriers are reduced.^19,20^ Vaccination programs may consider providing clear, balanced information on expected benefits, common mild adverse events, and postmarketing safety monitoring.^21,22^

Participants preferred community health care centers or stations over general and private hospitals, whereas no clear preference was observed regarding vaccination operating hours. The high relative importance of vaccination location was largely associated with the negative preference for private hospitals rather than by a difference between community health care centers and stations. This pattern suggests that the existing community-based delivery model is broadly aligned with older adults’ stated preferences. Community health care settings may be more familiar and prevention-oriented than hospitals, while the absence of a clear preference for extended operating hours may reflect greater scheduling flexibility among older adults, although this explanation was not directly evaluated.^23,24^

In contrast to findings from a younger US sample in which social media message features were associated with preference,^25^ telephone reminders and information from government or community organizations were preferred, suggesting that proactive contact and institutional trust remain important for this population. The preference for family-protection messages may reflect the relevance of intergenerational responsibility among older adults.^26^ These findings highlight proactive, personalized, and trust-based communication as practical targets for improving vaccination programs for older adults.

Preference heterogeneity was observed according to influenza vaccination history. Compared with those with prior vaccination, those who had never been vaccinated showed a greater tendency to opt out, were more sensitive to increases in the risk of mild adverse effects, and were less responsive to improvements in vaccine effectiveness. The exploratory latent class analysis provided complementary evidence that benefit-risk weighting was an important dimension of preference heterogeneity. Taken together, these findings suggest that prior vaccination experience may serve as a practical, observable marker of underlying differences in how older adults weigh vaccine benefits and risks.^27^ Thus, vaccination programs may need to tailor communication and service delivery to different behavioral drivers, rather than relying on a uniform strategy.^28,29^ For adults who have never been vaccinated, emphasizing higher effectiveness alone may be insufficient; transparent communication about mild adverse events, opportunities to discuss safety concerns, and counseling from trusted health care workers may be particularly important. At the same time, telephone outreach, information from government or community organizations, and messages emphasizing protection of family members were generally valued across vaccination history groups, supporting broad population-level strategies combined with more targeted safety-focused communication for never-vaccinated adults.

### Limitations

There are several limitations that should be considered in this study. First, as with all discrete choice experiments, stated preferences may not fully reflect practiced vaccination behavior. Second, participants were recruited using convenience sampling from 6 subdistricts in 2 Beijing districts and were not randomly selected. In addition, participants’ geographic proximity to vaccination sites was not assessed, which may have introduced selection bias and limited generalizability. However, the inclusion of subdistricts from Dongcheng and Changping Districts, which differ in urban development, health care resources, and service environments, provided some diversity in the study population. Third, because the number of eligible individuals invited or screened was not systematically recorded, a participation rate could not be calculated, and potential nonresponse bias could not be assessed. Additionally, although attribute selection was informed by literature review, expert consultation, and pretesting, other factors that may be associated with vaccination decisions were not included in the experimental design.

## Conclusions

In this discrete choice experiment of older adults in Beijing, influenza vaccination preferences were associated with vaccine effectiveness, perceived risk of mild adverse effects, community-based delivery, and trusted outreach. Vaccination history identified meaningful differences in benefit-risk weighting. These findings may inform the design and evaluation of population-wide and targeted vaccination strategies in publicly funded settings.

## Notes

Supplement 1. eTable 1. Reporting of the Survey-Based Discrete Choice Experiment According to AAPOR Disclosure Standards eTable 2. Reporting of the Survey-Based Discrete Choice Experiment According to DIRECT Checklist eTable 3. Rationale and Source for the Selection of Attributes and Levels eFigure. English Translation of an Example of Choice Set eMethods. Statistical Analysis eTable 4. Model Fit Statistics for Conditional Logit, Mixed Logit, and Latent Class Models eTable 5. Sensitivity Analysis of Mixed Logit Estimates Accounting for Recruitment Subdistrict Fixed Effects eTable 6. Utility-Equivalent Changes in Vaccine Effectiveness Required to Offset or Match Attribute-Level Preferences eTable 7. Pooled Mixed Logit Model Assessing Differences in Influenza Vaccination Preferences by Prior Vaccination History eTable 8. Exploratory Mixed Logit Estimates of Influenza Vaccination Preferences Stratified by Prior Influenza Vaccination History eTable 9. Exploratory Mixed Logit Estimates of Influenza Vaccination Preferences Stratified by sex eTable 10. Exploratory Mixed Logit Estimates of Influenza Vaccination Preferences Stratified by age group eTable 11. Exploratory Mixed Logit Estimates of Influenza Vaccination Preferences Stratified by Chronic Condition Status eTable 12. Exploratory Mixed Logit Estimates of Influenza Vaccination Preferences Stratified by Living Arrangement eTable 13. Preference Estimates From the 3-class Latent Class Logit Model

Supplement 2. Data Sharing Statement
