# Supplementary material for: Influenza Vaccination Preferences Among Adults Aged 60 Years and Older in Beijing, China
Source: JAMA Netw Open. 2026 Oct 12;9(10):e2638674. doi: 10.1001/jamanetworkopen.2026.38674 (PMC13659565; doi:10.1001/jamanetworkopen.2026.38674)
Supplement: Supplementary file 1 [file jamanetwopen-e2638674-s001.pdf]

Supplemental Online Content

Jia M, Liu Y, Zheng J, et al. Influenza vaccination preferences among adults aged 60 years and older in Beijing, China. *JAMA Netw Open.* 2026;9(10):e2638674. doi:10.1001/jamanetworkopen.2026.38674

eTable 1. Reporting of the Survey-Based Discrete Choice Experiment According to AAPOR Disclosure Standards

eTable 2. Reporting of the Survey-Based Discrete Choice Experiment According to DIRECT Checklist

eTable 3. Rationale and Source for the Selection of Attributes and Levels

eFigure 1. English Translation of an Example of Choice Set

eMethods. Statistical Analysis

eTable 4. Model Fit Statistics for Conditional Logit, Mixed Logit, and Latent Class Models

eTable 5. Sensitivity Analysis of Mixed Logit Estimates Accounting for Recruitment Subdistrict Fixed Effects

eTable 6. Utility-Equivalent Changes in Vaccine Effectiveness Required to Offset or Match Attribute-Level Preferences

eTable 7. Pooled Mixed Logit Model Assessing Differences in Influenza Vaccination Preferences by Prior Vaccination History

eTable 8. Exploratory Mixed Logit Estimates of Influenza Vaccination Preferences Stratified by Prior Influenza Vaccination History

eTable 9. Exploratory Mixed Logit Estimates of Influenza Vaccination Preferences Stratified by sex

eTable 10. Exploratory Mixed Logit Estimates of Influenza Vaccination Preferences Stratified by age group

eTable 11. Exploratory Mixed Logit Estimates of Influenza Vaccination Preferences Stratified by Chronic Condition Status

eTable 12. Exploratory Mixed Logit Estimates of Influenza Vaccination Preferences Stratified by Living Arrangement

eTable 13. Preference Estimates From the 3-class Latent Class Logit Model

© 2026 Jia M et al. *JAMA Network Open.*

This supplemental material has been provided by the authors to give readers additional information about their work.

© 2026 Jia M et al. *JAMA Network Open*.

eTable 1. Reporting of the survey-based discrete choice experiment according to AAPOR disclosure standards[1]

No.	Item	Page and paragraph
1	Data collection strategy	P7 of main text, "Study Design, Setting, Participants, and Ethics"
2	Who Sponsored the Research and Who Conducted It	Title page, and P18 of main text, "Acknowledgment"
3	Measurement Tools/Instruments	P8-9 of main text, "Survey Instrument"
4	Population Under Study	P7-8 of main text, "Study Design, Setting, Participants, and Ethics"
5	Method Used to Generate and Recruit the Sample	P7-8 of main text, "Study Design, Setting, Participants, and Ethics"
6	Method(s) and Mode(s) of Data Collection	P7-8 of main text, "Study Design, Setting, Participants, and Ethics"
7	Dates of Data Collection	P7-8 of main text, "Study Design, Setting, Participants, and Ethics"
8	Sample Sizes	P7-8 of main text, "Study Design, Setting, Participants, and Ethics"
9	How the Data Were Weighted	P11 of main text "Statistical Analyses"
10	How the Data Were Processed and Procedures to Ensure Data Quality	P9-10 of main text, "Attributes and levels", "Experimental Design"
11	A General Statement Acknowledging Limitations of the Design and Data Collection	P17 of main text, "Limitations"

[a]AAPOR: American Association for Public Opinion Research

© 2026 Jia M et al. *JAMA Network Open*.

eTable 2. Reporting of the survey-based discrete choice experiment according to DIRECT checklist[2]

Section	Item	Page and paragraph
Purpose and rationale	Describe the real-world context and decision-maker that the hypothetical choice context seeks to replicate or inform	P6-7 “Introduction” and p18 “Conclusions” of main text
	Provide a rationale for using a DCE to answer the research question	P6-7 of main text, “Introduction”
Attributes and levels	Describe how attributes and levels were derived	P9-10 of main text, “Attributes and levels”, eTable 3 in Supplement 1
	Provide the final list of attributes and levels	P22 of main text, “Table 1”
Experimental design	Report the number of alternatives per choice set and whether they were labelled or unlabelled	P10 of main text, “Experimental Design”
	Describe response options	P10 of main text, “Experimental Design”
	Describe the type of experimental design	P10 of main text, “Experimental Design”
	Describe which effects are identified in the design	P10 of main text, “Experimental Design”
	Describe the number of choice sets, blocks and choice sets per block	P10 of main text, “Experimental Design”
	Indicate how the experimental design was obtained	P10 of main text, “Experimental Design”
Survey design	Provide a sample choice set and the instructions and background information given to respondents	eFigure 1 in Supplement 1
	Report any randomisation	P10 of main text, “Experimental Design”
	Describe what was checked in piloting	P9-10 of main text, “Attributes and levels”
	Report whether information from the pilot was used to update the experimental design or survey design	P9-10 of main text, “Attributes and levels”
Sample and data collection	Report respondent inclusion/exclusion criteria	P8 of main text, “Study Design, Setting, Participants, and Ethics”
	Describe how data were collected	P8 of main text, “Study Design, Setting, Participants, and Ethics”
	Report the response rate or cooperation rate, if possible	P7 and P17 of main text, “Limitation”
	Report the final sample size and how the sample size was determined	P8 of main text, “Study Design, Setting, Participants, and Ethics”, P10, “Experimental Design”
	Describe respondent characteristics and representativeness of target population, if known	P12 of main text, “Participant characteristics”; P17 of main text, “Limitation”
Econometric analysis	Indicate coding of data (e.g. effects, dummy, continuous) including definitions	eMethods in Supplement 1
	Report whether any respondents were removed and why (e.g. suspected fraudulent responses, rationality tests)	P10 of main text, “Experimental Design”
	Provide the rationale for model choice and assumptions	P10-11 of main text, “Statistical analyses”, and eMethods in Supplement 1
	Report model specification	eMethods in Supplement 1
Reporting of results	Report the model performance, goodness of fit	eMethods and eTable 4 in Supplement 1
	Describe methods used for analysis of model results	P10-11 of main text, “Statistical analyses”, and eMethods in Supplement 1
	Report measures of precision for the output(s) of interest and how these were derived	eMethods in Supplement 1

[a] DIRECT: DIscrete choice experiment Reporting ChecklisT

© 2026 Jia M et al. *JAMA Network Open*.

© 2026 Jia M et al. *JAMA Network Open*.

eTable 3. Rationale and sources for the selection of attributes and levels

BeSD framework domain or vaccine profile	Attributes	Rationale for inclusion	Rationale for selection of levels	Sources
Practical issues	Location for vaccination	Vaccination location affects the convenience and accessibility of vaccination services and is directly modifiable through service-delivery arrangements.	The levels represented the principal types of settings in which influenza vaccination could be provided in Beijing.	Literature[3]; current influenza vaccination service models in Beijing; expert consultation; and pretesting
Practical issues	Operating hours	Operating hours may reduce temporal barriers and improve access to vaccination among older adults.	The levels represented commonly available service periods and potentially implementable extensions of vaccination service hours.	Literature[3, 4] ; local service practices; expert consultation; and pretesting
Social processes	Outreach method	Active reminders and invitations may help translate vaccination intentions into actual vaccination behavior.	The levels represented communication channels commonly used to provide vaccination information or reminders to older adults.	Literature[5, 6]; current community outreach practices; expert consultation; and pretesting
Social processes	Information source	The source of vaccination information may influence its perceived credibility and older adults' acceptance of vaccination.	The levels represented the major sources from which older adults may receive influenza vaccination information.	Literature[7, 8]; expert consultation; and pretesting
Thinking and feeling	Message content	The framing and content of vaccination messages may influence the perceived relevance, benefits, and motivation to receive vaccination.	The levels represented distinct but realistic benefit- and risk-focused messages that could be used in public health communication. The wording was refined during pretesting to improve clarity and minimize conceptual overlap.	Literature[9, 10]; expert consultation; and pretesting
Vaccine profile	Risk of mild side effects	Concerns about vaccine-related adverse effects are commonly reported barriers to influenza vaccination among older adults.	The levels represented plausible variations in the probability of mild and transient adverse effects following vaccination.	Literature[10, 11]; expert consultation; and pretesting
Vaccine profile	Vaccine effectiveness	Vaccine effectiveness has consistently been identified as an important determinant of influenza vaccine acceptance and choice.	The levels represented a broad but plausible range of effectiveness in preventing influenza infection.	Literature[10, 11]; expert consultation; and pretesting

[a] BeSD: Behavioral and Social Drivers

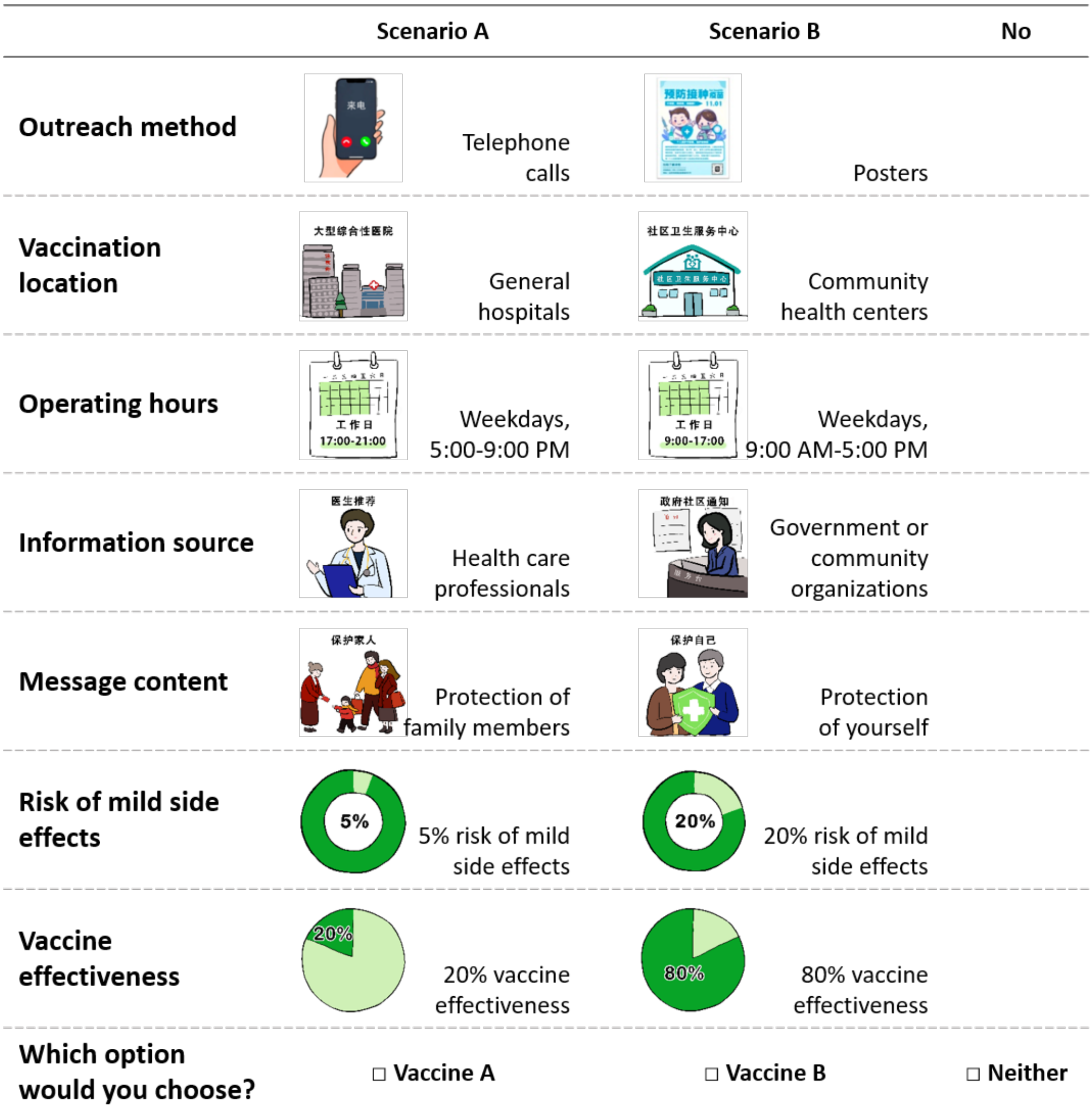

eFigure 1. English translation of an example of choice set

© 2026 Jia M et al. *JAMA Network Open*.

eMethods. Statistical analysis

Coding

Categorical attributes were dummy coded, with reference levels specified in the tables. The opt-out alternative was represented by an alternative-specific constant. Risk of mild side effects and vaccine effectiveness were modeled as continuous variables, ranging from 0.05 to 0.20 and from 0.20 to 0.80, respectively. Coefficients for these two attributes therefore represent changes in utility associated with a 1-unit change in the probability scale. For presentation in selected analyses, coefficients were rescaled to represent a 10-percentage-point change.

Conditional and mixed logit model

A conditional logit model was first estimated under the assumption of homogeneous preferences across participants. The choice-task identifier was specified as the grouping variable, and standard errors were clustered at the participant level to account for correlations among repeated choice tasks completed by the same participant. To account for repeated choices within participants and unobserved preference heterogeneity, a mixed logit model was subsequently estimated using simulated maximum likelihood. The choice set identifier was specified as the grouping variable, and the respondent identifier was specified as the panel variable to account for repeated choice observations from the same participant. To assess potential heterogeneity across recruitment subdistricts, a sensitivity analysis additionally incorporated subdistrict fixed effects through interactions between subdistrict indicators and the opt-out alternative-specific constant, allowing baseline vaccination choice to vary across the 6 recruitment subdistricts. The 5 interaction terms were jointly assessed using a Wald test.

In the mixed logit model, categorical attribute levels were initially considered for random specification based on theoretical relevance, preliminary model performance, and evidence of preference heterogeneity. The final model specified random parameters for selected attributes with substantive importance and improved model performance: telephone call and WeChat reminders, vaccination at community healthcare stations, general hospitals, and private hospitals, perceived risk of mild side effects, and vaccine effectiveness. These random parameters were assumed to follow normal distributions. The remaining coefficients, including the opt-out alternative-specific constant, short message reminders, operating hours, information source, and message content, were estimated as fixed parameters. The final mixed logit model was estimated using 1000 Halton draws. Estimated mean coefficients from the mixed logit model were used as the primary estimates for preference interpretation, relative attribute importance, trade-off analyses, and scenario simulations.

Relative attribute importance

Relative attribute importance was calculated using the range method. For each attribute, the difference between the highest and lowest estimated utility across the levels included in the experimental design was calculated. For risk of mild side effects and vaccine effectiveness, the utility range was calculated using the highest and lowest levels included in the DCE.

Trade-offs

Marginal rates of substitution were used to express utility changes associated with other attributes as utility-equivalent percentage-point changes in vaccine effectiveness. For each attribute level, the trade-off was calculated as the ratio of the attribute coefficient to the vaccine effectiveness coefficient and expressed as the equivalent percentage-point change in vaccine effectiveness. Confidence intervals for utility-equivalent changes in vaccine effectiveness were derived using the delta method. For a preferred attribute level, the estimate represented the increase in vaccine effectiveness that would generate an equivalent utility gain. For a nonpreferred attribute level, it represented the increase in vaccine effectiveness required to compensate for the associated utility loss. For the risk of mild side effects, trade-offs were calculated for 5-, 10-, and 15-percentage-point increases in perceived risk. These estimates represent trade-offs in stated preferences within the experimental design.

Scenario simulations

Scenario simulations were conducted to estimate the choice probability of hypothetical influenza vaccination profiles relative to the opt-out alternative. The simulations used the estimated mean coefficients from the mixed logit model. For each scenario, the systematic utility of the vaccination profile was calculated by summing the coefficients corresponding to the attribute levels included in that profile, whereas the utility of the opt-out alternative was represented by the estimated opt-out alternative-specific constant. The probability of choosing the vaccination profile rather than opting out was calculated using a binary logit transformation.

The baseline profile comprised vaccination at a community health care center during weekday daytime hours, poster-based outreach, information from social media, a message emphasizing prevention of influenza infection, a 20% perceived risk of mild side effects, and 20% vaccine effectiveness. Single-attribute scenarios changed one attribute level at a time while holding all other attributes at baseline levels. Sequential scenarios cumulatively added selected components related to vaccine profile,

© 2026 Jia M et al. *JAMA Network Open.*

practical issues, social processes, and thinking and feeling.

Changes in choice probabilities were expressed as percentage-point differences from the baseline scenario. Because probabilities were derived from a nonlinear logit transformation, the incremental contribution of each component in sequential scenarios depended on the order in which components were added. These estimates represent model-based stated-choice probabilities for hypothetical vaccination profiles.

Heterogeneity according to prior vaccination history

Prior influenza vaccination history was selected as the primary observed characteristic for assessing preference heterogeneity because it was directly measurable, approximately evenly distributed in the study population, and potentially actionable for intervention design. Participants were classified into 2 groups according to whether they reported ever receiving influenza vaccination before the survey. Mixed logit models were first estimated separately among participants with prior influenza vaccination and those who had never received influenza vaccination. The same attribute coding, reference levels, random-parameter specifications, and simulation settings used in the overall mixed logit model were applied to both stratified models.

A pooled mixed logit model was then fitted to formally assess differences between the 2 groups. The model included main effects for all attribute variables and interaction terms between never-vaccinated status and each model term, including the opt-out alternative-specific constant. Because prior vaccination history did not vary across alternatives within a choice task, a separate main effect for never-vaccinated status was not estimable. The main attribute effects represented coefficients among participants with prior vaccination, and each interaction term represented the difference between participants who had never been vaccinated and those with prior vaccination. Main attribute effects were retained as random parameters where applicable, whereas interaction terms were specified as fixed parameters to facilitate estimation and interpretation.

For the continuous attributes, risk of mild side effects and vaccine effectiveness were entered on a 0-to-1 scale in model estimation. For presentation, coefficients and between-group differences were rescaled to represent a 10-percentage-point increase. Statistical evidence of between-group heterogeneity was defined as a 95% CI for the interaction term that excluded 0.

Additional subgroup analyses

Additional subgroup analyses were conducted according to sex, age group, chronic condition status, and living arrangement. Mixed logit models were estimated separately within each subgroup using the same attribute coding, reference levels, random-parameter specifications, and simulation settings as the primary mixed logit model.

These analyses were considered exploratory because multiple subgroup comparisons were conducted and the subgroup variables were not selected as the primary basis for assessing preference heterogeneity. Results are presented descriptively in the Supplement. No adjustment was made for multiple comparisons.

Latent class analysis

Latent class logit models were estimated to identify subgroups with distinct preference patterns. Models with 2 to 4 classes were estimated. Model selection was based on log-likelihood, Akaike information criterion, Bayesian information criterion, class size, model stability, convergence, and substantive interpretability. Although the 4-class model showed improved information criteria, it produced an unstable or highly singular variance-covariance matrix; therefore, the 3-class model was selected as the final model based on the balance of statistical fit, model stability, class size, and interpretability. Individuals were assigned to the class with the highest posterior probability.

Software and procedures

Data management and quality control were conducted using SAS, version 9.4 (SAS Institute Inc). These procedures included importing the electronic questionnaire data, checking variable ranges and logical consistency, applying prespecified quality-control exclusions, recoding attribute levels and participant characteristics, deriving prior vaccination history and other subgroup variables, and restructuring the choice data into long format with 1 observation per alternative within each choice task. The resulting analysis-ready data set was exported for statistical modeling.

Statistical models were estimated using Stata, version 17.0 (StataCorp LLC). Conditional logit models were estimated using the Stata clogit command, with the choice-task identifier specified in the group() option and standard errors clustered at the participant level using the vce(cluster) option. Mixed logit models were estimated to account for repeated choices within respondents and preference heterogeneity, using the mixlogit command, with the choice-task identifier specified in the group() option, the respondent identifier specified in the id() option, and 1000 simulation draws specified using nrep(1000). The same mixed logit framework was used for stratified subgroup models and the pooled interaction model assessing heterogeneity according to prior vaccination history. Relative attribute

importance, marginal rates of substitution, scenario simulations, and latent class models were also conducted using Stata.
R, version 4.6.0 (R Foundation for Statistical Computing), was used only for data visualization and figure production. The ggplot2 package was used to construct the figures, and patchwork was used to assemble multipanel figures.

eTable 4. Model fit statistics for conditional logit, mixed logit, and latent class models

Models	LL (model)	AIC	BIC
**Conditional logit model**	-4004.08	8042.15	8168.29
**Mixed logit model**	-2882.22	5812.44	5990.53
**Latent class analyses**			
2-class	-2926.66	5923.33	6071.81
3-class (final model)	-2790.42	5686.84	5911.58
4-class[a]	-2667.07	5476.13	5777.33

* LL (model): Log-likelihood of fitted model
AIC: Akaike information criterion; BIC: Bayesian information criterion
[a] Variance matrix is nonsymmetric or highly singular.

eTable 5. Sensitivity Analysis of Mixed Logit Estimates Accounting for Recruitment Subdistrict Fixed Effects

	Sensitivity mixed logit results	
	**β (95% CI)**	***P* value**
**Opt-out**	0.37 (−0.49 to 1.24)	.40
**Outreach method (Reference: poster)**		
Short message	0.41 (0.10 to 0.71)	.01
Telephone call	1.07 (0.76 to 1.38)	<.001
WeChat	0.43 (0.15 to 0.71)	.01
**Location for vaccination (Reference: Community healthcare center)**		
Community healthcare station	0.07 (−0.17 to 0.30)	.58
General hospital	−1.13 (−1.44 to −0.82)	<.001
Private hospital	−4.58 (−5.26 to −3.90)	<.001
**Operating hours (Reference: 9 am-5 pm on weekdays)**		
5 pm-9 pm on weekdays	−0.12 (−0.36 to 0.12)	.34
9 am-12 pm on weekends	0.16 (−0.14 to 0.46)	.30
1 pm-5 pm on weekends	0.05 (−0.25 to 0.35)	.74
**Information source (Reference: social media)**		
Family members and friends	−0.16 (−0.40 to 0.08)	.20
Healthcare workers	0.26 (0.02 to 0.51)	.04
Government or community	0.67 (0.43 to 0.92)	<.001
**Message content (Reference: Getting vaccinated can prevent influenza infection)**		
Not getting vaccinated may result in severe infection	0.03 (−0.20 to 0.27)	.78
Getting vaccinated can protect family members	0.58 (0.34 to 0.83)	<.001
**Risk of mild side effects[a]**	−7.07 (−9.51 to −4.63)	<.001
**Vaccine effectiveness[a]**	1.68 (0.92 to 2.45)	<.001
**Subdistrict fixed effects[b]**		
Subdistrict 2 × opt-out	−0.52 (−1.64 to 0.59)	.36
Subdistrict 3 × opt-out	−1.08 (−2.10 to −0.06)	.04
Subdistrict 4 × opt-out	−0.77 (−1.66 to 0.12)	.09
Subdistrict 5 × opt-out	−1.96 (−2.95 to −0.98)	<.001
Subdistrict 6 × opt-out	0.32 (−0.56 to 1.20)	.48

[a] Risk of mild side effects and vaccine effectiveness were entered as continuous variables ranging from 0 to 1.

[b] Recruitment subdistrict was incorporated as a fixed effect through interactions between subdistrict indicators and the opt-out alternative-specific constant, with subdistrict 1 as the reference. The 5 subdistrict interaction terms were jointly significant (P < .001).

© 2026 Jia M et al. *JAMA Network Open*.

eTable 6. Utility-equivalent changes in vaccine effectiveness required to offset or match attribute-level preferences

	Equivalent change in vaccine effectiveness, percentage points (95% CI)[a]	P value
**Location for vaccination (Reference: Community healthcare center)**		
Community healthcare station	3.58 (-7.36 to 14.52)	.52
General hospital	52.63 (30.64 to 74.63)	<.001
Private hospital	221.35 (142.76 to 299.93)	<.001
**Operating hours (Reference: 9am-5pm on weekdays)**		
5pm-9pm on weekdays	5.25 (-6.08 to 16.57)	.36
9am-12pm on weekends	8.85 (-4.31 to 22.02)	.19
1pm-5pm on weekends	4.15 (-8.72 to 17.01)	.53
**Outreach method (Reference: Poster)**		
Short message	13.28 (-1.39 to 27.95)	.08
Telephone call	50.06 (28.77 to 71.34)	<.001
WeChat	19.54 (5.01 to 34.06)	.01
**Information source (Reference: social media)**		
Family members and friends	0.83 (-9.78 to 11.44)	.88
Healthcare workers	12.28 (-0.61 to 25.17)	.06
Government or community	36.71 (20.54 to 52.89)	<.001
**Message content (Reference: Getting vaccinated can prevent influenza infection)**		
Not getting vaccinated may result in severe infection	1.09 (-9.73 to 11.91)	.84
Getting vaccinated can protect family members	23.58 (10.79 to 36.38)	<.001
**Risk of mild side effects**		
5-percentage-point increase	13.02 (6.05 to 19.99)	<.001
10-percentage-point increase	26.04 (12.10 to 39.97)	<.001
15-percentage-point increase	39.06 (18.15 to 59.96)	<.001

[a]Values represent utility-equivalent percentage-point changes in vaccine effectiveness derived from mixed logit coefficients. Estimates should be interpreted as trade-offs in stated preferences rather than actual changes in vaccine efficacy.

© 2026 Jia M et al. *JAMA Network Open*.

eTable 7. Pooled mixed logit model assessing differences in influenza vaccination preferences by prior vaccination history

Attributes and levels	Prior vaccination(n=262), β (95% CI)	Never vaccinated (n=252), β (95% CI)	Differences, never vs prior vaccination, β (95% CI)	P for interaction[a]
**Opt-out**	-0.46 (-1.12 to 0.20)	0.61 (-0.11 to 1.33)	1.07 (0.11 to 2.03)	.03
**Location for vaccination (Reference: Community healthcare center)**				**.04**
Community healthcare station	0.01 (-0.30 to 0.32)	0.14 (-0.21 to 0.50)	0.14 (-0.33 to 0.61)	.57
General hospital	-1.41 (-1.79 to -1.02)	-0.69 (-1.11 to -0.28)	0.71 (0.16 to 1.26)	.01
Private hospital	-4.87 (-5.63 to -4.11)	-4.12 (-4.91 to -3.33)	0.75 (-0.09 to 1.60)	.08
**Operating hours (Reference: 9am-5pm on weekdays)**				**.47**
5pm-9pm on weekdays	-0.26 (-0.57 to 0.05)	0.08 (-0.28 to 0.44)	0.34 (-0.13 to 0.81)	.16
9am-12pm on weekends	0.15 (-0.22 to 0.52)	0.25 (-0.16 to 0.66)	0.10 (-0.46 to 0.65)	.73
1pm-5pm on weekends	0.09 (-0.27 to 0.45)	0.12 (-0.29 to 0.53)	0.03 (-0.51 to 0.58)	.91
**Outreach method (Reference: Poster)**				**.17**
Short message	0.21 (-0.19 to 0.60)	0.34 (-0.11 to 0.78)	0.13 (-0.46 to 0.72)	.67
Telephone call	0.97 (0.58 to 1.36)	1.16 (0.73 to 1.60)	0.20 (-0.38 to 0.78)	.51
WeChat	0.62 (0.26 to 0.98)	0.23 (-0.18 to 0.64)	-0.39 (-0.94 to 0.15)	.16
**Information source (Reference: Social media)**				**.53**
Family members and friends	0.01 (-0.29 to 0.31)	0.04 (-0.31 to 0.38)	0.03 (-0.42 to 0.48)	.90
Healthcare workers	0.41 (0.08 to 0.74)	0.07 (-0.30 to 0.44)	-0.34 (-0.83 to 0.16)	.18
Government or community	0.77 (0.47 to 1.08)	0.75 (0.39 to 1.10)	-0.03 (-0.49 to 0.44)	.91
**Message content (Reference: Getting vaccinated can prevent influenza infection)**				**.48**
Not getting vaccinated may result in severe infection	0.02 (-0.28 to 0.32)	-0.05 (-0.40 to 0.30)	-0.07 (-0.53 to 0.38)	.75
Getting vaccinated can protect family members	0.40 (0.09 to 0.71)	0.59 (0.24 to 0.94)	0.19 (-0.28 to 0.66)	.43
**Risk of mild side effects**	-3.14 (-6.01 to -0.26)	-8.05 (-11.40 to -4.70)	-4.91 (-9.29 to -0.54)	**.03**
**Vaccine effectiveness**	3.34 (2.40 to 4.29)	1.56 (0.51 to 2.61)	-1.78 (-3.13 to -0.43)	**.01**

[a] Overall P values for categorical attributes were obtained from joint Wald tests of all interaction terms within the corresponding attribute.

© 2026 Jia M et al. *JAMA Network Open*.

eTable 8. Exploratory mixed logit estimates of influenza vaccination preferences stratified by prior influenza vaccination history

	Prior vaccination		Never vaccinated	
	β (95% CI)	P value	β (95% CI)	P value
**Opt-out**	-0.28 (-0.9 to 0.35)	.39	0.43 (-0.35 to 1.21)	.28
**Location for vaccination (Reference: Community healthcare center)**				
Community healthcare station	0.02 (-0.26 to 0.29)	.92	0.12 (-0.27 to 0.52)	.54
General hospital	-1.31 (-1.7 to -0.91)	<.001	-0.68 (-1.09 to -0.27)	<.001
Private hospital	-4.58 (-5.45 to -3.72)	<.001	-4.31 (-5.28 to -3.34)	<.001
**Operating hours (Reference: 9am-5pm on weekdays)**				
5pm-9pm on weekdays	-0.26 (-0.56 to 0.04)	.09	0.14 (-0.24 to 0.52)	.48
9am-12pm on weekends	0.15 (-0.2 to 0.51)	.39	0.27 (-0.17 to 0.71)	.23
1pm-5pm on weekends	0.04 (-0.3 to 0.38)	.81	0.11 (-0.34 to 0.55)	.64
**Outreach method (Reference: Poster)**				
Short message	0.25 (-0.12 to 0.61)	.19	0.36 (-0.13 to 0.85)	.15
Telephone call	0.88 (0.51 to 1.26)	<.001	1.34 (0.86 to 1.82)	<.001
WeChat	0.62 (0.27 to 0.97)	<.001	0.24 (-0.19 to 0.67)	.27
**Information source (Reference: social media)**				
Family members and friends	0.01 (-0.28 to 0.29)	.97	0.06 (-0.3 to 0.42)	.75
Healthcare workers	0.37 (0.05 to 0.68)	.02	0.11 (-0.29 to 0.5)	.59
Government or community	0.74 (0.44 to 1.03)	<.001	0.84 (0.46 to 1.22)	<.001
**Message content (Reference: Getting vaccinated can prevent influenza infection)**				
Not getting vaccinated may result in severe infection	0.05 (-0.23 to 0.33)	.73	-0.05 (-0.43 to 0.33)	.81
Getting vaccinated can protect family members	0.42 (0.12 to 0.71)	.01	0.66 (0.27 to 1.05)	<.001
**Risk of mild side effects**	-2.66 (-5.37 to 0.05)	.05	-10.04 (-14.19 to -5.89)	<.001
**Vaccine effectiveness**	3.01 (2.17 to 3.86)	<.001	0.82 (-0.7 to 2.34)	.29

© 2026 Jia M et al. *JAMA Network Open*.

eTable 9. Exploratory mixed logit estimates of influenza vaccination preferences stratified by sex

	Male		Female	
	β (95% CI)	P value	β (95% CI)	P value
**Opt-out**	-0.65 (-1.45 to 0.15)	.11	0.43 (-0.21 to 1.07)	.19
**Location for vaccination (Reference: Community healthcare center)**				
Community healthcare station	-0.08 (-0.44 to 0.29)	.69	0.09 (-0.21 to 0.39)	.54
General hospital	-0.99 (-1.44 to -0.54)	<.001	-1.16 (-1.54 to -0.78)	<.001
Private hospital	-4.37 (-5.41 to -3.32)	<.001	-4.92 (-5.82 to -4.02)	<.001
**Operating hours (Reference: 9am-5pm on weekdays)**				
5pm-9pm on weekdays	-0.07 (-0.45 to 0.32)	.73	-0.14 (-0.44 to 0.17)	.39
9am-12pm on weekends	0.36 (-0.08 to 0.8)	.11	0.08 (-0.28 to 0.45)	.65
1pm-5pm on weekends	0.42 (-0.03 to 0.87)	.07	-0.06 (-0.41 to 0.29)	.75
**Outreach method (Reference: Poster)**				
Short message	0.07 (-0.41 to 0.54)	.79	0.43 (0.05 to 0.81)	.03
Telephone call	0.81 (0.35 to 1.26)	<.001	1.24 (0.85 to 1.63)	<.001
WeChat	0.42 (0.00 to 0.84)	.05	0.47 (0.11 to 0.83)	.01
**Information source (Reference: social media)**				
Family members and friends	-0.08 (-0.45 to 0.28)	.66	0.07 (-0.22 to 0.36)	.65
Healthcare workers	0.25 (-0.16 to 0.67)	.24	0.27 (-0.04 to 0.59)	.09
Government or community	0.83 (0.44 to 1.22)	<.001	0.74 (0.43 to 1.04)	<.001
**Message content (Reference: Getting vaccinated can prevent influenza infection)**				
Not getting vaccinated may result in severe infection	-0.11 (-0.48 to 0.25)	.55	0.07 (-0.23 to 0.37)	.65
Getting vaccinated can protect family members	0.42 (0.04 to 0.80)	.03	0.54 (0.24 to 0.85)	<.001
**Risk of mild side effects**	-4.12 (-7.93 to -0.31)	.03	-6.24 (-9.19 to -3.28)	<.001
**Vaccine effectiveness**	2.20 (1.16 to 3.25)	<.001	2.37 (1.38 to 3.37)	<.001

© 2026 Jia M et al. *JAMA Network Open.*

eTable 10. Exploratory mixed logit estimates of influenza vaccination preferences stratified by age group

	≤70 years old		>70 years old	
	**β (95% CI)**	**P value**	**β (95% CI)**	**P value**
**Opt-out**	-0.17 (-0.89 to 0.56)	.65	0.16 (-0.53 to 0.85)	.65
**Location for vaccination (Reference: Community healthcare center)**				
Community healthcare station	0.32 (-0.02 to 0.65)	.06	-0.18 (-0.5 to 0.14)	.27
General hospital	-0.82 (-1.19 to -0.44)	<.001	-1.35 (-1.8 to -0.91)	<.001
Private hospital	-3.88 (-4.64 to -3.12)	<.001	-5.59 (-6.77 to -4.41)	<.001
**Operating hours (Reference: 9am-5pm on weekdays)**				
5pm-9pm on weekdays	-0.12 (-0.46 to 0.22)	.49	-0.14 (-0.47 to 0.2)	.42
9am-12pm on weekends	0.22 (-0.17 to 0.61)	.27	0.1 (-0.3 to 0.5)	.62
1pm-5pm on weekends	0.04 (-0.34 to 0.42)	.83	0.13 (-0.27 to 0.54)	.52
**Outreach method (Reference: Poster)**				
Short message	0.63 (0.2 to 1.07)	.01	-0.06 (-0.49 to 0.36)	.77
Telephone call	1.01 (0.59 to 1.43)	<.001	1.14 (0.69 to 1.59)	<.001
WeChat	0.42 (0.02 to 0.82)	.04	0.41 (0.04 to 0.79)	.03
**Information source (Reference: social media)**				
Family members and friends	-0.10 (-0.43 to 0.22)	.53	0.12 (-0.2 to 0.43)	.46
Healthcare workers	0.30 (-0.05 to 0.64)	.10	0.21 (-0.15 to 0.58)	.25
Government or community	0.86 (0.51 to 1.21)	<.001	0.68 (0.35 to 1.01)	<.001
**Message content (Reference: Getting vaccinated can prevent influenza infection)**				
Not getting vaccinated may result in severe infection	-0.03 (-0.36 to 0.29)	.83	0.02 (-0.31 to 0.35)	.92
Getting vaccinated can protect family members	0.57 (0.22 to 0.91)	<.001	0.41 (0.08 to 0.75)	.02
**Risk of mild side effects**	-7.42 (-10.69 to -4.16)	<.001	-3.35 (-6.61 to -0.09)	.04
**Vaccine effectiveness**	2.74 (1.67 to 3.82)	<.001	2.24 (1.19 to 3.30)	<.001

© 2026 Jia M et al. *JAMA Network Open*.

eTable 11. Exploratory mixed logit estimates of influenza vaccination preferences stratified by chronic condition status

	With chronic condition		Without chronic condition	
	β (95% CI)	P value	β (95% CI)	P value
**Opt-out**	-0.16 (-1.05 to 0.73)	.72	0.20 (-0.40 to 0.80)	.51
**Location for vaccination (Reference: Community healthcare center)**				
Community healthcare station	0.06 (-0.39 to 0.51)	.79	0.04 (-0.22 to 0.3)	.78
General hospital	-0.85 (-1.31 to -0.39)	<.001	-1.20 (-1.57 to -0.82)	<.001
Private hospital	-3.28 (-4.25 to -2.31)	<.001	-5.04 (-5.91 to -4.18)	<.001
**Operating hours (Reference: 9am-5pm on weekdays)**				
5pm-9pm on weekdays	0.12 (-0.31 to 0.55)	.58	-0.22 (-0.50 to 0.05)	.12
9am-12pm on weekends	0.21 (-0.28 to 0.7)	.39	0.18 (-0.15 to 0.52)	.28
1pm-5pm on weekends	-0.33 (-0.82 to 0.16)	.18	0.22 (-0.10 to 0.54)	.18
**Outreach method (Reference: Poster)**				
Short message	0.49 (-0.04 to 1.02)	.07	0.28 (-0.08 to 0.63)	.12
Telephone call	1.41 (0.87 to 1.96)	<.001	0.93 (0.58 to 1.28)	<.001
WeChat	0.18 (-0.31 to 0.67)	.48	0.60 (0.27 to 0.93)	<.001
**Information source (Reference: social media)**				
Family members and friends	-0.02 (-0.44 to 0.39)	.91	0.03 (-0.23 to 0.3)	.81
Healthcare workers	0.10 (-0.33 to 0.52)	.65	0.25 (-0.05 to 0.56)	.10
Government or community	0.41 (-0.01 to 0.84)	.06	0.92 (0.64 to 1.20)	<.001
**Message content (Reference: Getting vaccinated can prevent influenza infection)**				
Not getting vaccinated may result in severe infection	0.20 (-0.21 to 0.62)	.34	-0.09 (-0.36 to 0.18)	.50
Getting vaccinated can protect family members	0.72 (0.28 to 1.16)	<.001	0.41 (0.13 to 0.70)	<.001
**Risk of mild side effects**	-7.79 (-11.72 to -3.85)	<.001	-4.14 (-7.02 to -1.26)	.01
**Vaccine effectiveness**	2.29 (0.86 to 3.72)	<.001	2.08 (1.03 to 3.13)	<.001

© 2026 Jia M et al. *JAMA Network Open*.

eTable 12. Exploratory mixed logit estimates of influenza vaccination preferences stratified by living arrangement

	Living alone		Living with family		Living in a nursing home	
	β (95% CI)	P value	β (95% CI)	P value	β (95% CI)	P value
**Opt-out**	-1.8 (-3.53 to -0.07)	.04	0.07 (-0.55 to 0.69)	.82	0.61 (-0.41 to 1.63)	.24
**Location for vaccination (Reference: Community healthcare center)**						
Community healthcare station	0.78 (0.03 to 1.53)	.04	0.20 (-0.08 to 0.47)	.16	-0.39 (-0.93 to 0.15)	.16
General hospital	-0.63 (-1.66 to 0.40)	.23	-1.05 (-1.40 to -0.71)	<.001	-1.28 (-1.93 to -0.64)	<.001
Private hospital	-2.93 (-4.39 to -1.46)	<.001	-4.80 (-5.59 to -4.01)	<.001	-4.56 (-5.99 to -3.13)	<.001
**Operating hours (Reference: 9am-5pm on weekdays)**						
5pm-9pm on weekdays	0.24 (-0.49 to 0.97)	.51	-0.13 (-0.42 to 0.16)	.39	-0.17 (-0.67 to 0.33)	.50
9am-12pm on weekends	0.33 (-0.57 to 1.23)	.48	0.25 (-0.09 to 0.59)	.15	0.00 (-0.61 to 0.61)	.99
1pm-5pm on weekends	0.55 (-0.30 to 1.40)	.21	0.14 (-0.20 to 0.47)	.43	-0.16 (-0.73 to 0.42)	.60
**Outreach method (Reference: Poster)**						
Short message	0.17 (-0.79 to 1.13)	.73	0.34 (-0.04 to 0.71)	.08	0.19 (-0.42 to 0.8)	.55
Telephone call	0.91 (-0.06 to 1.88)	.07	1.22 (0.85 to 1.59)	<.001	0.71 (0.04 to 1.38)	.04
WeChat	-0.42 (-1.29 to 0.44)	.34	0.51 (0.17 to 0.85)	<.001	0.62 (0.02 to 1.22)	.04
**Information source (Reference: social media)**						
Family members and friends	-0.08 (-0.79 to 0.64)	.84	0.06 (-0.22 to 0.33)	.68	-0.10 (-0.60 to 0.40)	.69
Healthcare workers	-0.08 (-0.88 to 0.72)	.84	0.36 (0.05 to 0.67)	.02	0.09 (-0.45 to 0.64)	.74
Government or community	0.75 (-0.02 to 1.52)	.06	0.81 (0.52 to 1.10)	<.001	0.82 (0.32 to 1.33)	<.001
**Message content (Reference: Getting vaccinated can prevent influenza infection)**						
Not getting vaccinated may result in severe infection	-0.34 (-1.09 to 0.40)	.37	-0.02 (-0.3 to 0.26)	.88	0.00 (-0.49 to 0.49)	>.99
Getting vaccinated can protect family members	-0.34 (-1.12 to 0.44)	.39	0.60 (0.30 to 0.90)	<.001	0.62 (0.13 to 1.12)	.01
**Risk of mild side effects**	-6.33 (-12.87 to 0.20)	.06	-6.71 (-9.61 to -3.81)	<.001	-0.94 (-5.43 to 3.55)	.68
**Vaccine effectiveness**	2.17 (-0.18 to 4.52)	.07	1.88 (0.93 to 2.83)	<.001	2.20 (1.01 to 3.39)	<.001

© 2026 Jia M et al. *JAMA Network Open*.

eTable 13. Preference estimates from the 3-class latent class logit model

Attributes and levels	Class 1	Class 2	Class 3
**Opt-out**	4.13	-1.77	0.55
**Location for vaccination (Reference: Community healthcare center)**			
Community healthcare station	-0.23	0.31	-0.15
General hospital	-0.39	-1.06	0.26
Private hospital	-3.68	-3.09	-0.97
**Operating hours (Reference: 9am-5pm on weekdays)**			
5pm-9pm on weekdays	-0.48	0.04	0.12
9am-12pm on weekends	-0.23	0.28	0.13
1pm-5pm on weekends	-0.27	0.28	-0.21
**Outreach method (Reference: Poster)**			
Short message	0.34	0.46	0.19
Telephone call	1.35	0.92	0.7
WeChat	-0.11	0.84	0.17
**Information source (Reference: Social media)**			
Family members and friends	0.55	-0.19	-0.15
Healthcare workers	-0.08	0.07	0.01
Government or community	1.31	0.42	0.16
**Message content (Reference: Getting vaccinated can prevent influenza infection)**			
Not getting vaccinated may result in severe infection	0.09	-0.09	-0.05
Getting vaccinated can protect family members	0.65	0.27	0.17
**Risk of mild side effects**	-0.16	-4.38	-4.97
**Vaccine effectiveness**	1.93	0.55	5.23
**Estimated class share**	30.50%	41.60%	27.90%

© 2026 Jia M et al. *JAMA Network Open*.

References:

1. American Association for Public Opinion Research. AAPOR Code of Professional Ethics & Practice (April 2021). 2021. https://aapor.org/standards-and-ethics/disclosure-standards/#1667933142558-b5099a52-a1eb. August 1, 2026

2. Ride J, Goranitis I, Meng Y, LaBond C, Lancsar E. A Reporting Checklist for Discrete Choice Experiments in Health: The DIRECT Checklist. *PHARMACOECONOMICS.* 2024;42(10):1161-1175. doi:10.1007/s40273-024-01431-6

3. Eiden AL, Barratt J, Nyaku MK. A review of factors influencing vaccination policies and programs for older adults globally. *HUM VACC IMMUNOTHER.* 2023;19(1):2157164. doi:10.1080/21645515.2022.2157164

4. Jiang B, Li M, Dai P, et al. Employees' seeking preference towards influenza vaccination in organization: A discrete choice experiment in China. *HELIYON.* 2024;10(9):e30432. doi:10.1016/j.heliyon.2024.e30432

5. Thomas RE, Lorenzetti DL. Interventions to increase influenza vaccination rates of those 60 years and older in the community. *The Cochrane database of systematic reviews.* 2018;5(5):CD005188. doi:10.1002/14651858.CD005188.pub4

6. Johansen ND, Vaduganathan M, Bhatt AS, et al. Electronic nudges to increase influenza vaccination uptake in Denmark: a nationwide, pragmatic, registry-based, randomised implementation trial. *The Lancet.* 2023;401(10382):1103-1114. doi:10.1016/S0140-6736(23)00349-5

7. Jiang M, Li P, Yao X, et al. Preference of influenza vaccination among the elderly population in Shaanxi province, China. *HUM VACC IMMUNOTHER.* 2021;17(9):3119-3125. doi:10.1080/21645515.2021.1913029

8. Bonner KE, Chyderiotis S, Sicsic J, et al. What motivates adults to accept influenza vaccine? An assessment of incentives, ease of access, messaging, and sources of information using a discrete choice experiment. *SSM - Population Health.* 2023;22:101384. doi:10.1016/j.ssmph.2023.101384

9. Johansen ND, Vaduganathan M, Bhatt AS, et al. Electronic nudges for sustained influenza vaccination uptake in older adults: the nationwide randomized NUDGE-FLU-2 trial. *NAT MED.* 2024;30(11):3142-3149. doi:10.1038/s41591-024-03202-4

10. Hou Z, Guo J, Lai X, et al. Influenza vaccination hesitancy and its determinants among elderly in China: A national cross-sectional study. *VACCINE.* 2022;40(33):4806-4815. doi: 10.1016/j.vaccine.2022.06.063

11. Liao Q, Ng TWY, Cowling BJ. What influenza vaccination programmes are preferred by healthcare personnel? A discrete choice experiment. *VACCINE.* 2020;38(29):4557-4563. doi:10.1016/j.vaccine.2020.05.012

© 2026 Jia M et al. *JAMA Network Open.*
