# Supplementary material for: Influenza Vaccination Preferences Among Adults Aged 60 Years and Older in Beijing, China
Source: JAMA Netw Open. 2026 Oct 12;9(10):e2638674. doi: 10.1001/jamanetworkopen.2026.38674 (PMC13659565; doi:10.1001/jamanetworkopen.2026.38674)
Supplement: Supplementary file 2 [file jamanetwopen-e2638674-s002.pdf]

Data Sharing Statement

Jia. Influenza Vaccination Preferences Among Adults Aged 60 Years and Older in Beijing, China. *JAMA Netw Open*. Published October 12, 2026.
doi:10.1001/jamanetworkopen.2026.38674

Data
Data available: Yes
Data types: Deidentified participant data, Data dictionary
How to access data: Deidentified participant data and the data dictionary will be available from the corresponding author (fengluzhao@cams.cn) upon reasonable request. Investigators who provide a methodologically sound proposal for achieving the aims of an approved study may gain access to the data after approval by the study investigators and completion of a data use agreement.
When available: With publication

Supporting Documents
Document types: None

Additional Information
Who can access the data: Researchers whose proposed use of the data has been approved
Types of analyses: For any scientific purpose
Mechanisms of data availability: After approval of a proposal and signed data access agreement
